# Formation and Controlled Drug Release Using a Three-Component Supramolecular Hydrogel for Anti-*Schistosoma Japonicum* Cercariae

**DOI:** 10.3390/nano6030046

**Published:** 2016-03-09

**Authors:** Yibao Li, Lei Zhu, Yulan Fan, Yayun Li, Linxiu Cheng, Wei Liu, Xun Li, Xiaolin Fan

**Affiliations:** Key Laboratory of Organo-pharmaceutical Chemistry, Gannan Normal University, Ganzhou 341000, China; zhul@gnnu.cn (L.Z.); fanyl@gnnu.cn (Y.F.); liyy@gnnu.cn (Y.L.); chenglx@gnnu.cn (L.C.); liuw@gnnu.cn (W.L.); wuyq@gnnu.cn (X.L.)

**Keywords:** hydrogel, drug release, long-term release time, anti-cercariae

## Abstract

A novel three-component supramolecular hydrogel based on riboflavin, melamine and amino acid derivatives were constructed for controlled release of pesticides, Niclosamide derivatives. The formation of hydrogel may be attributed to self-assemble via hydrogen bonding and π–π interaction, which have been researched via scanning electron microscopy (SEM) and Fourier transform infrared (FT-IR) spectra. The rheological experiments showed that the hydrogel materials and drug-loaded hydrogel all demonstrated good mechanical strength and high stability. Further experimental results indicated that the drug-loaded hydrogels show large drug loadings, long-term release time and relatively higher efficiency to anti-cercariae in the water environment.

## 1. Introduction

Recently, supramolecular hydrogels have attracted intense research interest, particularly due to their possible practical applications in controlled drug delivery [1,2,3], biomedicine [4], cell culture [5], mechano-responsive sensor materials [6], and pollutant capture and removal [7]. The hydrogelators, amino acids, sugars and steroids can be extracted easily from organisms; therefore, supramolecular hydrogels perform several advantages over the currently polymer gels [8]. In the past ten years, many multicomponent supramolecular hydrogels based on biomolecules have been reported [9,10,11]. For biological applications, a constant challenge has been finding environmentally friendly molecules to use for controlled drug release [12]. Many small gelator molecules could overcome this problem, since the diversity of functionality is derived from biocompatible components and held together by noncovalent forces [13].

Supramolecular hydrogels are formed through spontaneous aggregation of gelator molecules to structure three-dimensional self-assembled fibrillar networks (SAFINs) [14]. The driving force is mainly derived from intermolecular non-covalent interactions such as hydrogen bonding, π–π stacking, donor–acceptor interactions, metal coordination, solvophobic forces and van der Waals interactions [15,16]. During the multicomponent hydrogel self-sorting process, hydrogen bonding was able to contribute a great amount of driving forces [17]. Recently, two-component hydrogels of riboflavin and melamine, which are suitable for biomedicine and drug delivery systems, have been reported. The formation of the hydrogels could be attributed to the hydrogen bonding interaction between the carbonyl oxygen of riboflavin and the hydrogen atoms of melamine to produce the complexes [18,19,20,21,22].

In addition, π–π stacking has been generally known to be the intermolecular interaction of the molecules containing an aromatic ring. As an excellent provider of the strong π–π stacking, perylene diimides (PDIs) as versatile ingredients have an enormous application range, such as molecular switches [23], organic lightemitting diodes [24], photoreactive thin films [25], light-harvesting arrays [26], dye lasers, and solar cells [27]. Furthermore, amino acid and its derivatives were, due to their good biocompatibility and environmental friendliness, have been used to build biological materials [28,29], as the favorable gelling agent molecules, amino acid and its derivatives have been used to generate hydrogels with the applications of biological and sensor [30,31].

Schistosomiasis remains a primary, hazardous, rambunctious chronic infections disease in many tropical areas [32]. Shockingly, there are 280,000 deaths annually, 200 million people have been infected, 779 million are at risk of infection, and more than 20 million individuals have experienced high morbidity, all due to schistosomiasis, [33,34,35]. The cercarial stage has been the only cause of infection, and the most fragile phase in the life cycle of schistosome [36]. Because more than 98% of cercariae float on water, a thin cercaricide film that can float on the water surface has been able to kill cercariae [37]. There are some drugs that have been able to effectively prevent *Schistosoma japonicum* cercariae, such as Niclosamide, praziquantel, plants destroyed cercaria drugs, and so on. Unfortunately, these drugs have shortcomings: They have low solubility in water, they are difficult to extract, and the cost is high [38]. To improve the floatation performance of pesticides, the new-type Niclosamide derivatives have been developed by Fan and colleagues [39]. However, in a natural environment, the anti-cercarial results of the Niclosamide derivatives have not been ideal because the pesticides quickly lose their effectiveness in the water soon after they are applied, [40]. Therefore, there is great significance in improving the action time for cercaricide.

Herein, the supramolecular three-component hydrogel based on melamine, riboflavin and amino acid derivatives was constructed via self-assembled behavior. To further study the material, mechanical properties, drug release systems and anti-cercarial ability were investigated.

## 2. Results and Discussion

**Scheme 1:**
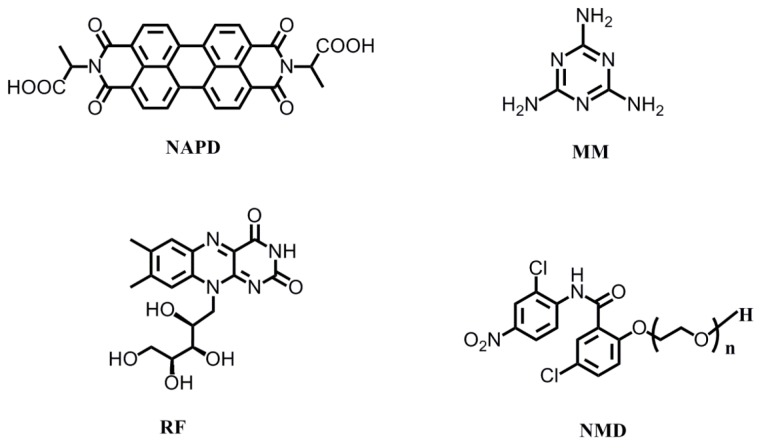
Chemical structures of *N*,*N*′-dialanine-3,4,9,10-tetracarboxylic diimide (NAPD); melamine (MM); riboflavin (RF) and Niclosamide derivatives (NMD).

The chemical structures of *N*,*N*′-dialanine-3,4,9,10-tetracarboxylic diimide (NAPD), riboflavin (RF), melamine (MM) and Niclosamide derivatives (NMD) are shown in Scheme 1. The structure of NAPD demonstrates that it is composed of two parts: the perylene with pronounced capabilities of π–π stacking and terminal carboxy as remarkable donors and receptors of hydrogen bonds in the gelation process. The structure of RF and MM both contain planar benzene ring structures, which are beneficial for the π-stacking process. The amidogens of MM and hydroxyls of RF participate in the self-assembly process through the H-bonds. We tested the gelation ability of compounds NAPD with RF and MM in a complex solution (THF/H_2_O = 1:1) in a typical test-tube experiment. It was found that the NAPD/MM/RF (1/2/2) system (0.77% *w/v*) was able to generate a rufous and steady gel under ultrasound at 25 °C. In particular, ultrasound was necessary for the gel formation under the same conditions. It was possible for the intermolecular forces, such as hydrogen bonding and π–π stacking, to obtain enough energy from ultrasound to form network structures.

**Figure 1:**
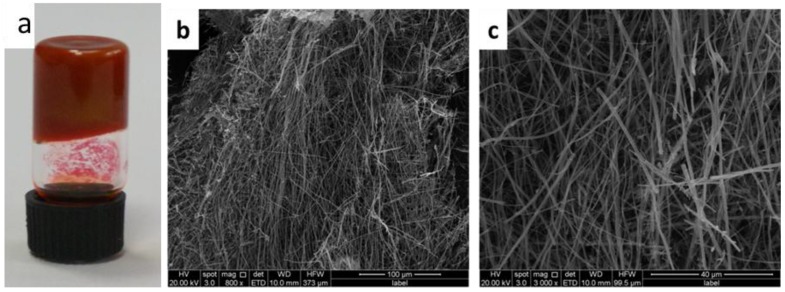
(**a**) Photo of the gel; scanning electron microscope (SEM) images of the samples (**b**) for NAPD/MM/RF ([NAPD] = 5 g/L) in larger scale; (**c**) for NAPD/MM/RF in small scale.

Xerogel samples were observed with scanning electron microscopy (SEM) to investigate the self-assembly structure of gels. The samples were prepared via freeze-drying in a vacuum to protect the microstructures. As shown in Figure 1, fibrous structures were the major constituents in the xerogels of the NAPD/MM/RF (1/2/2) system. Further, the inter-twisted and inter-locked fibers based on the wispy fibrous structure formed the three-dimensional porous network structure, (Figure 1b,c). It was clearly observed that there were many gaps in the network structure, which were a great help in locking the solvent and pharmaceutical molecules.

**Figure 2:**
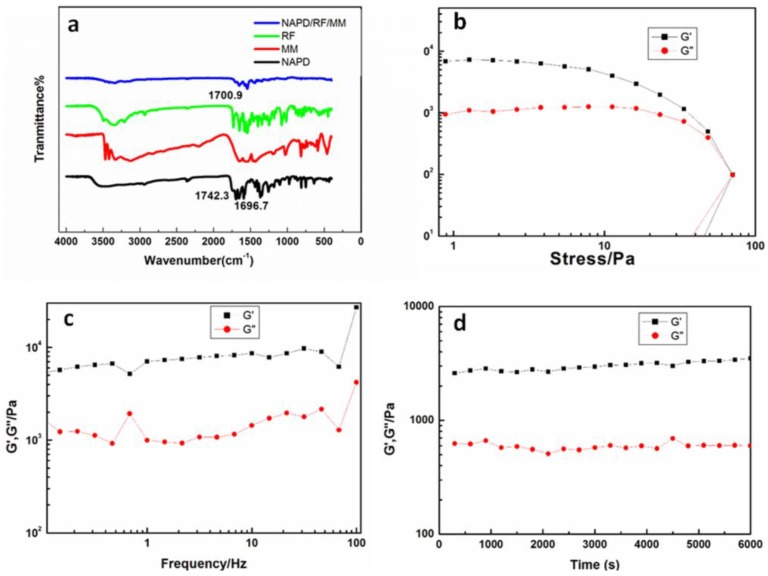
(**a**) Fourier transform infrared spectroscopy (FT-IR) spectra diluted with KBr for a NAPD/RF/MM = 1/2/2 xerogel (**blue**), NAPD (**black**), RF (**green**), MM (**red**); (**b**) strain sweep for NAPD/RF/MM gel at 1.5% *w*/*v* concentration; (**c**) frequency sweep of the NAPD/MM/RF system at the strain of 0.1%; (**d**) time sweep of the NAPD/MM/RF gel at the strain of 30%.

As we know, Fourier transform infrared (FT-IR) spectroscopy is an effective method of investigating intermolecular interactions in the gelation. It could provide some important evidence for understanding the hydrogen bonding interactions in the gel formation. In Figure 2a, the bands of pure NAPD at 1742 cm^−1^ and 1696 cm^−1^ correspond to the free carboxyl groups of NAPD, and peaks were shifted to 1700 cm^−1^, which is indicative of hydrogen bonding C=O stretching frequency. The results revealed the formation of the hydrogen bonding in the course of gelation [41,42]. It indicated that the hydrogen bonding between MM, RF and NAPD make great contributions to the formation of the gel.

As an important property, the mechanical performance of a gel material has a great influence on its practical application [35]. To explore the dynamic mechanical properties of the gel, the rheology was characterized by a rheometer. According to Figure 2b, in the linear viscoelastic region (LVR), the dynamic storage modulus G′ reached 10,000 Pa, and it was larger than the corresponding dynamic loss modulus G′′. As shown in Figure 2c, the results of the frequency sweep for hydrogels revealed that the storage modulus G′ was larger than the loss modulus G′′ over the investigated oscillating frequency range, and the difference between G′ and G′′ was nearly unchanged. Figure 2d showed a time sweep at a strain of 30%, indicating that the viscoelasticity maintained invariability over 6000 s. The above results suggest that this extremely stable hydrogel has excellent mechanical properties.

**Figure 3:**
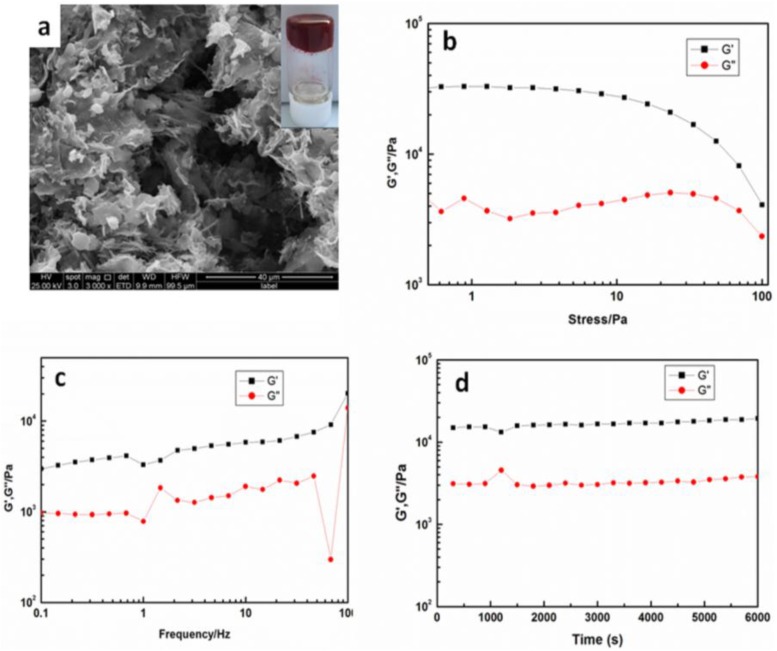
(**a**) SEM images of the xerogels for NAPD/MM/RF/NMD hydrogel ([NAPD] = 5 g/L, [NMD] = 10 g/L) (Insert: Photo of the gel); (**b**) Strain sweep for NAPD/RF/MM/NMD gel at above concentration; (**c**) Frequency sweep of the NAPD/RF/MM/Drugs gel at the strain of 0.1%; (**d**) Time sweep of the NAPD/RF/MM/NMD gel at the strain of 30%.

Drug content is an important parameter for evaluating the properties of the drug-loaded materials. As shown in Figure 3a, it was able to reach the maximum value of the drug content of NMD (200 mg) by 1 mL NAPD/RF/MM gel (1.5% *w*/*v*) at the molar ratio of 1/2/2 without destroying gel formation. The SEM images show that the drugs were loaded into the intrinsic three-dimensional porous network structure, and that fibrous structures could still be found in the network structure. It has been suggested that the NMD molecule was loaded into the hydrogel materials and formed a stable structure. Next, we further performed the rheology experiments to explore its stability. As plotted in Figure 3b, the value of storage modulus G′ was over 10^4^ Pa and was larger than the corresponding dynamic loss modulus G′′ in the strain sweep, which suggests that the hydrogel was a viscoelastic material. According to Figure 3c, the G′ is always larger than G′′ from 0.01 Hz to 10 Hz and their difference basically remains unchanged, which means that the gels were not sensitive to the exoteric force. The time sweep showed that the viscoelasticity of hydrogels did not change in 6000 s in Figure 3d. This suggests that drug molecules were completely wrapped in the hydrogel, and that the stable hydrogel system has good mechanical properties.

**Figure 4:**
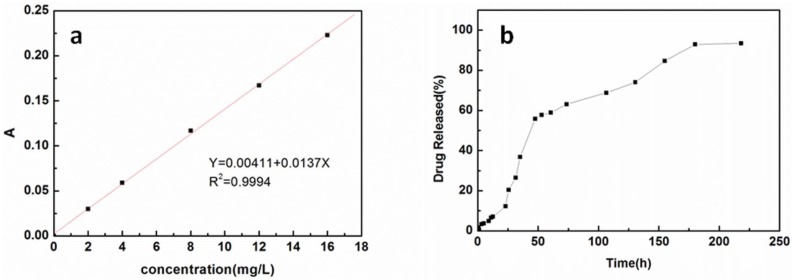
(**a**) Standard curves of ultraviolet (UV) absorption: Niclosamide derivatives at 326.5 nm; (**b**) Drug release behavior of NMD from the NAPD/MM/RF = 1/2/2 hydrogel at 25 °C in water.

Figure 4 showed the release profiles of Niclosamide derivatives from the GP/MM/RF system in water. Figure 4a shows the ultraviolet (UV) absorption standard curves of Niclosamide derivatives at 326.5 nm. The unknown experimental concentration of NMD can be calculated based on the formula (*Y* = 0.00411 + 0.0137*X*) based on the standard curves. Figure 4b shows the release profiles of NMD from the NAPD/MM/RF = 1/2/2 system. It can be observed that about 95% of NMD was released from the hydrogel over 200 h. Additionally, there was a burst release during the first 50 h, after which the release rate reduced significantly. The results indicate that Niclosamide derivatives can smoothly release into water from the NAPD/MM/RF = 1/2/2 system. This drug release system can satisfy the objective of an efficient and long-term cercaricide in a water environment.

Further, the anti-cercarial ability of the release system was investigated. The anti-cercarial activity experiment was conducted in a released solution at different release times. As shown in Table 1, the effect of anti-cercarial activity was prominent. Firstly, cercariae was incubated in the solution for 1 h, and the mortality rate of cercariae was nearly 0. However, after 60 min, the cercariae began to die; the mortality rate reached more than 80% over 120 min. Further, with added release time, the mortality rate of cercariae can almost reach 100% in 60 min. Therefore, the hydrogel release system NAPD/MM/RF/NMD maintained commendable bioactivity against cercariae in the water environment.

## 3. Materials and Methods

### 3.1. Materials

*N*,*N*-dialanine-3,4,9,10-tetracarboxylic diimide (NAPD) and the Niclosamide derivatives (NMD) were synthesized and characterized in a laboratory. Double-distilled water (DDW) was used in all experiments. All chemicals and reagents were commercially available and used without further processing.

### 3.2. Synthesis of NAPD

Under an atmosphere of nitrogen, perylene-3,4,9,10-tetracarboxylic dianhydride (392 mg, 1.0 mmol), L-alanine (0.178 g, 2.0 mmol) and 2.0 g imidazole were mixed in a 50-mL flask. Then, the commixtures were stirred at 120 °C for 6 h, and the resulting emulsion was cooled at 90 °C. 25 mL of ethanol were added and stirred for 2 h. Subsequently, the mixture was cooled down at room temperature and filtered. Then, the residue was dissolved in H_2_O, and 1.0 moL/L of HCl was further added dropwise until the water became acidic. After precipitation, the precipitate was filtered and washed with H_2_O. The product was dried at room temperature, and mulberry powder was produced (yield: 0.481 g, 90%). The structure and purity of the product were confirmed by ^1^H NMR, FT-IR and MS. IR (KBr): 1255.4, 1401.6, 1590.2 cm^−1^ (C=C), 1365.5, 1436.5 cm^−1^ (–CH_3_), 1655.3, 1696.7, 1742.3 cm^−1^ (C=O), 2940.5 cm^−1^ (C–H), 3443.3 cm^−1^ (O–H); ^1^H NMR (DMSO, 400 MHz, ppm): 8.01–8.03 (d, 8 H), 5.52–5.56 (m, 2 H), 1.66–1.68 (d, 6 H); MALDI-TOF-MS: calcd for C_30_H_18_N_2_O_8_, 534.0.

### 3.3. Characterization

Nuclear magnetic resonance (NMR) spectra were recorded on an ULTRASHIELD 400 (Bruker, Zürich, Switzerland) spectrometer at 400 MHz at room temperature. Electrospray ionization mass spectra ((ESI-MS)) were obtained by Micromass LCTTM system (Altringham, UK). The FT-IR spectra were carried out using KBr discs on an AVATAR 360 FT-IR spectrophotometer (Nicolet, Madison, WI, USA) at room temperature. UV absorption spectra were performed using a UV-1800 UV-Vis spectrophotometer (Shimadzu, Tokyo, Japan). The morphology of these samples was observed via scanning electron microscopy (FEI, QUANTA 450, Hillsboro, OR, USA) with an accelerating voltage of 20.0 kV. The SEM samples were prepared by placing a drop of gel on a flat surface of a cylindrical copper substrate. Then, the hydrogel was dried via freeze-drying for 3 h. Following that, the samples were coated with gold using a MSP-1S magnetron sputter (Japan) coater (Tokyo, Japan). Rheological properties were measured using a HAAKE RheoStress 6000 rheometer (Offenburg, Germany) with plate geometry (plate diameter, 35 mm). Frequency sweep measurements were carried out on freshly formed gels over a range from 0.01 to 10 Hz at 25 °C. Strain sweep was performed at a strain amplitude of 1%. Time sweep measurements were performed at a frequency of 6.28 rad·s^−1^ and a strain of 30%.

### 3.4. Gelation Test

The gelation test of *N*,*N*-dialanine-3,4,9,10-tetracarboxylic diimide (NAPD), riboflavin (RF) and melamine (MM) in a complex solution (THF/H_2_O = 1:1) were performed. To prepare different hydrogel systems at various NAPD:RF:RF molar ratios, generous amounts of NAPD, RF and MM were weighed in glass tubes, and comparable amount of double distilled water was then added after an addition of 0.5 mL of tetrahydrofuran (THF). They were endured for 2 h with ultrasonic vibration at room temperature. The results showed that the NAPD:RF:RF hydrogel system at 1/2/2 molar ratio can form steadily and does not flow when the tube is inverted. It was discovered that the minimum gelation concentration for this system is 0.77% (*w*/*v*).

### 3.5. Drug Loading Test

The Niclosamide derivatives (NMD) were used to test the release property of this controlled release system. To investigate the drug loading, different qualities of the NMD were added to 1 mL of a complex solution (THF/H_2_O = 1:1) with 10 mg gels of NAPD, MM and RF at the molar ratio of 1/2/2. Then, the compounds were sustained with ultrasonic treatment for 2 h at room temperature. The formation of the gels was observed to investigate the maximum drug loadings of this kind of supramolecular gels material.

### 3.6. In Vitro Release

The release of Niclosamide derivatives (NMD) was obtained from the measurement of absorbance at 326.5 nm and a standard curve, and expressed as follows: NMD release (%) = NMD released/NMD total × 100%. The 2-mL hydrogel sample with 10-mg gels of NAPD, MM and RF at the molar ratio of 1/2/2, which was loaded with 20 mg of NMD, was dried via freeze-drying in a vacuum for 6 h. The drug release research was performed by immersing the xerogels sample in a 150-mL portion of water at 25 °C and stirring. 0.5 mL of the solution was taken out from the glass bottle at the planned time, and the equal fresh water was then added to insure the same total solution volume. The concentrations of the NMD were measured by the quantitative determination method at 326.5 nm.

### 3.7. Anti-Cercarial Activity Experiments

According to the *in vitro* release test, a 300-μL solution was removed from the glass in a 96-hole edition at different release times (1 h, 2 h, 5 h, 24 h, 200 h, in this order). *S. japonicum* cercariae were acquired from the water surface in a conical flask, and the infected *O. hupensis* was soaked with bright lighting for 2 h. The cercariae were removed from the holes, including the above solution, at different release times. The activity of the cercariae was observed every 30 min via biological microscopy.

## 4. Conclusions

In this paper, a three-component supramolecular hydrogel was successfully fabricated, which was composed of amino acid derivatives, melamine and riboflavin. The FT-IR experimental results revealed that the driving forces are the hydrogen bonding between carboxyls. The hydrogels exhibited excellent mechanical strength and large drug loadings of Niclosamide derivatives. Significantly, the release time of the Niclosamide derivatives can reach 10 days, and the release rate can nearly reach 100%. Finally, the results of the anti-cercarial ability experiment showed that the release system exhibits good efficiency in killing cercariae in a water environment.

## Figures and Tables

**Table 1:** The mortality rate of *Schistosoma japonicum* cercariae for the hydrogel release system NAPD/RF/MM/Drug ([NAPD] = 5 g/L, [Drug] = 10 g/L) (25 °C).

Release Time (h)	Mortality (%)			
	30 min	60 min	90 min	120 min
1	0	23.5	41.2	82.4
2	6.67	20	80	100
5	85.7	100	100	100
24	100	100	100	100
200	100	100	100	100
black	0	0	0	0

^1^ All experiments were performed at least in triplicate.

## Abbreviations

NAPD: *N*,*N′*-dialanine-3,4,9,10-tetracarboxylic diimide
RF: riboflavin
MM: melamine
NMD: Niclosamide derivatives
